# Ego-resilience and health-related quality of life after acquired Upper Limb Amputation

**DOI:** 10.1371/journal.pone.0352208

**Published:** 2026-06-25

**Authors:** Lauren A. Trent, Anna Zanotto

**Affiliations:** 1 University of Kansas Medical Center, Kansas City, KS, USA; 2 Advanced Arm Dynamics, Inc., Redondo Beach, CA, USA; Brunel University London, UNITED KINGDOM OF GREAT BRITAIN AND NORTHERN IRELAND

## Abstract

**Purpose:**

The purpose of this study was to examine the relationships between demographic variables, ego-resilience and health-related quality of life (HRQoL) for individuals with upper limb amputations. As HRQoL continues to be an important measure of rehabilitative success, determining universal factors which correlate to and may predict HRQoL becomes more important in the clinical space.

**Methods:**

A sample of 90 previously administered outcomes from patients at a national upper limb prosthetic provider in the United States were gathered. The outcome measure, the Wellness Inventory, captured patient-reported data to screen for mental health status including ego-resilience, PTSD, depression, coping mechanisms. Scores from the Orthotics and Prosthetics Users’ Survey (OPUS) HRQoL as well as the Ego-Resilience Scale (ER89) were utilized in this study, as well as pertinent demographic data. Comparative analyses were conducted on the data gathered.

**Results:**

HRQoL and Ego-Resilience scores were analyzed alongside demographic factors: gender (77.8% male), age at time of amputation (mean age 38, SD = 12.9), level of amputation, ethnicity and marital status. Correlational analysis showed positive relationship between ego-resilience and HRQoL (ρ = 0.332, p = .002). Simple linear regression analysis found a significant relationship between ethnicity and HRQoL (β = 4.237, p = .047), and ego-resilience and HRQoL (β = 0.910, p=<.001). The multiple linear regression model identified ego-resilience and ethnicity as predictors for HRQoL (adjusted R² = .129, F = 7.576, p=<.001).

**Conclusion:**

Based on the findings in this study, ego-resilience has been identified as a significant predicting factor for HRQoL. Higher ego-resilience score and trait likely will result in higher HRQoL scores. Understanding of how demographic variables, such as ethnicity, may directly or indirectly impact HRQoL can also be beneficial in the recovery process.

## Introduction

An estimated 5.6 million people are living with some form of limb loss or limb difference in the United States, with approximately 17% annual amputations are upper limb [1]. The leading cause of upper limb amputation (ULA) is trauma, and its effect on an individual can be significantly more complex and life-altering than other causes of amputation [2]. Despite the majority of all trauma-related amputations being of the upper extremity, and due to the larger proportion of individuals with lower limb amputation, the research available which examines both tend to include the majority of participants with lower limb amputations [2,3]. The effects of upper limb amputation on quality of life remain an understudied topic.

After undergoing an ULA, individuals must surmount a litany of obstacles, as it directly affects functional daily tasks such as self-care, home management and occupational responsibilities, as well as self image, self confidence, and health related quality of life (HRQoL) [2]. HRQoL has been widely studied in the lower limb amputee population, due to its higher prevalence, but has not been studied in depth in the upper limb amputee population [3]. One study suggests that severity of injury as well as location (proximal versus distal) had a significant impact on HRQoL for the individual [4]. Factors such as age at amputation, level of amputation, and resiliency may affect a person’s perceived quality of life and have significant bearing on their functional and mental health. While there is a large gap in the literature for the upper limb amputee population, several of these factors have been studied in the lower limb amputee population: a study in 2019 found that older individuals (75 + years) with amputation demonstrated a lower HRQoL due to increased physical limitations [5]. Another study found that higher levels of amputation reported an increase in functional difficulty, especially when combined with more complex prostheses; however this study also demonstrated a significant difference between adolescents and adults in the ability to cope with this physical change [6].

Quality of life (QoL) is a multi-faceted concept that incorporates many factors including physical, emotional and psychological statuses, as well as social and environmental influences [7]. According to the World Health Organization (WHO), QoL is how a person views their own living conditions, taking into account the cultural, social, and environmental context they live in, as well as how these factors connect to their personal goals, expectations, and concerns [8]. QoL can also be examined within the context of health, including general health, physical symptoms, emotional well-being, cognitive functioning to existential and spiritual aspects [7]. In the lower limb amputee population, QoL is becoming a widely recognized outcome of rehabilitation, but has been more regularly utilized to compare interventions, different populations, and different treatment options [9]. Despite its documented importance in rehabilitation, health-related quality of life (HRQoL) has not been extensively studied in the upper limb amputee population.

Ego-resilience is conceptualized as a stable personality trait inherent in the self, reflecting an individual’s dynamic flexibility in impulse control and their ability to adapt to changing circumstances [10]. Unlike process-based resilience, which can be transient or heavily dependent on external social and environmental factors, ego-resilience is viewed as a foundational characteristic essential for understanding personal emotion, behavior, and motivation [11,12]. This internal capacity for self-regulation has been found to directly impact a person’s ability to cope with significant health changes, thereby influencing overall well-being and health-related quality of life (HRQoL). Furthermore, higher levels of ego-resilience are associated with a lower prevalence of psychological illnesses, such as depression and anxiety, serving as a key factor in a person’s perception of their recovery [13]. While ego-resilience has been studied as a mediator of quality of life in populations with diagnoses such as cancer, stroke, and multiple sclerosis, its specific role in the upper limb amputee population has not yet been thoroughly examined [11,14]. Over the past decade, mental health research for the upper limb amputee population in the United States has been minimal, despite the majority of limb amputations being acquired after birth or early childhood [1]. Having a potentially significant physical change not only affects functional ability, but also self-image and confidence [15]. A study by Kearns et al. (2018) found that level of amputation has significant impact on functional impairments as well as psychological implications. There have been several studies which show that demographic factors, such as age and gender, can greatly impact resiliency and perceived HRQoL when dealing with stressful events [13,16,17]. While function of the extremity can be quantifiable, the quality of life and psychological status of the patient is not as easily measured. There are many potential determinants of these aspects, including sociodemographic factors, injury etiology and health-related factors [4,9].

In an attempt to quantify this aspect of health, the Wellness Inventory (WI) outcome measure was developed by Advanced Arm Dynamics, a company specializing in upper limb prosthetic care and rehabilitation, to gather data on the mental health status of upper limb amputees in their care [18]. The WI screens for several factors, including perceived quality of life, resilience, depression, post-traumatic anxiety and pain. The purpose of this study is to examine the relationship between demographic factors, ego- resilience and HRQoL.

## Methods

### Participants

Data collection occurred at seven regional Advanced Arm Dynamics clinics within the United States which specialize in upper limb prosthetic rehabilitation between 2019 and 2025. The demographic information and outcome measure data collected was a part of the clinics’ standard of care, and each participant consented to allow for use of their deidentified data. On April 24, 2025, deidentified retrospective data were accessed through a research partnership between the University of Kansas Medical Center and Advanced Arm Dynamics. The lead author, who serves in a clinical leadership role at the organization, facilitated the extraction of deidentified records to ensure compliance with privacy standards. A representative sample of 90 individuals with upper limb amputations were identified utilizing this previously collected data. For the data to be included in the study, the participant had to meet the following inclusion criteria: have an acquired upper limb amputation, be 18 years of age or older at the time of outcome measure administration, be able to understand the WI directions and content, and had consented to have deidentified data included in research.

### Procedures

Ethical approval for collection of data was originally approved by the WCG IRB in 2019 (9/9/2019, Study 1264637), then received secondary approval for analysis by the University of Kansas Medical Center IRB. The data was collected utilizing the Wellness Inventory (WI) between the years 2019 and 2024. Informed consent was obtained from all individual participants included in the original data collection. The WI consists of portions of multiple measures used to screen for psychological barriers for prosthetic use: resilience (Ego-Resilience Scale), health-related quality of life (OPUS), pain (Short Form Health Survey), depression (VA Depression screening tool), alcohol use (Alcohol Use Disorders Identification Test – Consumption), drug use/misuse (two clinically developed, face-valid questions), and posttraumatic anxiety (Primary Care PTSD screen) [18–24].

### Measures

This study utilizes a subset of data from a larger clinical surveillance dataset collected via the Wellness Inventory (WI). While the WI serves as a comprehensive screening tool used in standard prosthetic care, this analysis focuses specifically on the instruments measuring ego-resilience and health-related quality of life (HRQoL).

The Ego-Resilience Scale (ER89) was developed to measure ego-resiliency through self-report ratings [10]. This scale treats resilience as a trait, rather than a transient behavior, that is “the individual ability to dynamically and appropriately self-regulate, allowing highly resilient people to adapt more quickly to changing circumstances” [12]. The ER89 was initially created based on questionnaire responses from previously identified resilient individuals, through a study comparing the relationship between ego-resiliency and IQ [10]. This 14 item questionnaire utilizes a 4-point Likert scale (1 = does not apply at all, 2 = applies slightly, if at all, 3 = applies somewhat, 4 = applies very strongly). For this scale, high scores correspond with high levels of resiliency. The original scale had a coefficient alpha reliability (Cronbach’s α) of 0.76 (with a score closer to 1.0 being desired to demonstrate internal consistency of the items). A later validation in 2004, investigating the generalization of this scale found a Cronbach’s α of 0.72 across 188 participants [25].

The Orthotics and Prosthetics Users’ Survey (OPUS) was initially developed to fill a need for evaluating orthotic and prosthetic (O&P) clinical outcomes [26]. The HRQoL subscale from OPUS was developed to subjectively measure the patient’s perceived quality of life, which, at that time, was not being consistently measured for the O&P patient population. This 23 item scale utilizes a two 5-point Likert scales: a frequency scale (all of the time, most of the time, some of the time, a little of the time and none of the time) and an extent of agreement scale (not at all, slightly, somewhat, quite a bit, extremely), with four of the 23 items being reverse scored. In initial development, the Rasch analysis was used to evaluate the instrument effectiveness in measure the construct of HRQoL. This yielded a separation index of 2.74 (with a score over 2.0 being desired), and a reliability of 0.88 (which is interpreted the same as Cronbach’s α, with a score closer to 1.0 being desired) [26].

### Statistical analysis

Prior to conducting the linear regression, the data were screened to ensure the assumptions of the model were met. Linearity was confirmed via scatterplots of the variables. The independence of residuals was verified using the Durbin-Watson statistic. Homoscedasticity was assessed through visual inspection of a plot of standardized residuals against standardized predicted values. Finally, multicollinearity was ruled out as Variance Inflation Factor (VIF) values for all predictors were well below the threshold of 10.

Initial descriptive analysis was performed on demographic data including age at time of amputation, age at administration of measure, gender, level of amputation, marital status and ethnicity. Demographic data was described utilizing means, proportions, ranges and standard deviations. Correlational analyses were performed to investigate the associations between demographics, ego-resilience scores and HRQoL scores. Spearman’s rho was selected for all correlational analyses to maintain methodological consistency across the study, as several demographic variables (e.g., level of amputation, marital status) exhibited non-normal distributions. This conservative, non-parametric approach ensured that comparisons between psychological scores and non-normal demographic data remained valid. Finally, to examine variables that could predict HRQoL, multiple linear regression analysis was conducted, step-wise selection modeling, a method for systematically identifying relevant predictor variables, was used for significant variable selection [27]. All analyses were conducted utilizing IBM SPSS Statistics (Version 29).

## Results

In the sample of 90 participants, 77.8% were male and 22.2% were female. The age at time of amputation and age at time of administration were measured in years and mean, SD and range were reported. Amputation level was broken into the following commonly utilized subcategories: Digit(s), Partial Hand, Wrist Disarticulation, Transradial, Elbow Disarticulation, Transhumeral, Shoulder Disarticulation. The majority of participants reported Digit amputation level (58.9%) Ethnicity was reported as Asian, Black, Hispanic, White or Other. The majority of participants reported as White (81.1%). Marital status was reported as Single, Married, Divorced or Widowed, with the majority being Married (55.6%). For details on demographic characteristics of the sample, see Table 1.

**Table 1:** Demographic characteristics and clinical descriptive statistics for participants with acquired upper limb amputation (n = 90). This table summarizes the participant sample by gender, age, ethnicity, marital status, and amputation level. It also provides the means and standard deviations for the primary study variables, including pain, depression, ego-resilience, and health-related quality of life (HRQoL).

Characteristics	
*Gender, % (n)*	
*Male*	77.8% (70)
*Female*	22.2% (20)
*Age at time of amputation (y)*	
*Mean (SD)*	38 (12.9)
*Range*	17–69
*Age at time of data collection*	
*Mean (SD)*	39.9 (12.7)
*Range*	18–75
*Amputation Level, % (n)*	
*Digit(s)*	58.9% (53)
*Partial Hand*	17.8% (16)
*Wrist Disarticulation*	0% (0)
*Transradial*	13.3% (12)
*Elbow Disarticulation*	0% (0)
*Transhumeral*	8.9% (8)
*Shoulder Disarticulation*	1.1% (1)
*Ethnicity, % (n)*	
*Asian*	2.2% (2)
*Black*	3.3% (3)
*Hispanic*	11.1% (10)
*White*	81.1% (73)
*Other*	2.2% (2)
*Marital Status, %(n)*	
*Single*	38.9% (35)
*Married*	55.6% (50)
*Divorced*	4.4% (4)
*Widowed*	1.1% (1)
*Pain*	
*Mean (SD)*	1.57 (1.09)
*Range*	0–4
*Depression*	
*Mean (SD)*	1.34 (1.49)
*Range*	0–4
*Alcohol Use Disorders Identification Test – Consumption (AUDIT-C)*	
*Mean (SD)*	2.81 (2.76)
*Range*	0–10
*Drug Misuse*	
*Mean (SD)*	1.33 (0.373)
*Range*	0–2
*Primary Care – PTSD screen (PC – PTSD)*	
*Mean (SD)*	1.51 (1.57)
*Range*	0–4
*Ego-resilience*	
*Mean (SD)*	44.6 (5.18)
*Range*	29–56
*Orthotic and Prosthetic Users’ Survey – HRQoL (OPUS – HRQoL)*	
*Mean (SD)*	55.3 (13.1)
*Range*	25–88

Upon initial analysis, it was found that while the scores for ego-resilience and HRQoL followed a normal distribution, the other factors did not. Correlational analysis showed significant positive relationship (α = 0.05) between ego-resilience and HRQoL (Spearman’s rho = 0.332, p = 0.002), as well as age at amputation and age at administration of the measures (Spearman’s rho = 0.939, p = < 0.001) (see Table 2). To ensure statistical rigor, a post-hoc Pearson correlation was also conducted on these normally distributed parameters, confirming the strength and significance of the relationship (r = 0.36, p < .001). Kruskal-Wallis tests were done to compare between group medians for significant differences. There was only one significant difference found, between age at administration of the measure and marital status (χ2 = 12.34, p = 0.006). All other comparisons of groups were not found to be significantly different (see Table 3). Chi-square analysis showed significant dependence between marital status and ethnicity (χ2 = 25.5, p = 0.012). No significant dependencies were found in pairwise comparison between the remaining categorical variables: gender, level of amputation, marital status and ethnicity.

**Table 2:** Spearman’s rho correlation matrix for participant demographics, ego-resilience, and health-related quality of life. This matrix displays the non-parametric correlation coefficients used to evaluate the strength and direction of associations between age, gender, ego-resilience, and HRQoL scores. Statistical significance is indicated where p < 0.05.

		Age at Amputation	Age at Administration	Gender	Ego-Resilience	HRQoL
Age at Amputation	Spearman’s rho	—				
	p-value	—				
Age at Administration	Spearman’s rho	0.939	—			
	p-value	< .001*	—			
Gender	Spearman’s rho	−0.035	−0.063	—		
	p-value	0.743	0.553	—		
Ego -Resilience	Spearman’s rho	0.078	0.045	0.025	—	
	p-value	0.463	0.677	0.813	—	
HRQoL	Spearman’s rho	0.099	0.084	0.041	0.332	—
	p-value	0.353	0.432	0.703	0.002*	—

Note. *statistically significant (α = 0.05).

**Table 3:** Kruskal-Wallis tests for group differences in ego-resilience and health-related quality of life by demographic category. This grouped table presents the results of non-parametric comparisons used to determine if scores for the primary psychological constructs differed significantly based on categorical groupings such as marital status, gender, and ethnicity.

Kruskal-Wallis (Marital Status)			
	χ²	df	p
Resilience	4.09	3	0.251
HRQoL	4.00	3	0.261
Age at Amp	8.48	3	0.037
Age at Admin	12.34	3	0.006*
Months since Amp	5.13	3	0.162
Kruskal-Wallis (Gender)			
	**χ²**	**df**	**p**
Resilience	0.0568	1	0.812
HRQoL	0.1664	1	0.683
Age at Amp	0.1090	1	0.741
Age at Admin	0.3566	1	0.550
Months since Amp	0.3760	1	0.540
Kruskal-Wallis (Ethnicity)			
	**χ²**	**df**	**p**
Resilience	3.16	4	0.532
HRQoL	7.07	4	0.132
Age at Amp	2.29	4	0.682
Age at Admin	2.85	4	0.583
Months since Amp	3.84	4	0.428

* statistically significant, α = *0.05*.

Utilizing simple linear regression analysis, ego-resilience was found to be a statistically significant predictor of HRQoL (α = 0.05, β = 0.910, t = 3.626, p = < 0.001). Ethnicity was also found to be a significant predictor of HRQoL (α = 0.05, β = 4.237, t = 2.019, p = 0.047). When analyzed together in multivariate linear regression analysis, ego-resilience scores and ethnicity predictors model was statistically significant (α = 0.05, p = < .001), however the contribution of these predictors differed, with resilience being found to be the only statistically significant predictor (see Table 4). Diagnostic checks confirmed that the regression assumptions were satisfied. The Durbin-Watson statistic was 2.02, indicating an absence of autocorrelation and confirming the independence of residuals. Furthermore, VIF values were 1.05 for all predictors, demonstrating no significant multicollinearity between ego-resilience and ethnicity. When utilizing stepwise selection modeling, the statistically significant model utilized on ego-resilience as the predictor. This selection modeling excluded gender, age at time of amputation, age at time of outcome measure administration, amputation level, ethnicity and marital status from the multiple predictor model based on lack of significance in affecting the model outcome.

**Table 4:** Summary of simple and multiple linear regression models predicting health-related quality of life. This table outlines the predictive power of ego-resilience and ethnicity on HRQoL outcomes. It includes standardized coefficients (β), t-statistics, and the adjusted R2 for each model, with diagnostic notes regarding the Durbin-Watson and Variance Inflation Factor (VIF) values.

Models	β	t	Sig	Adj R-square	F	Sig
1 Resilience	.360	3.626	<.001	.120	13.145	<.001
2 Ethnicity	.210	2.019	.047	.033	4.076	.047
3 Resilience	.330	3.260	.002	.129	7.576	<.001
Ethnicity	.139	1.370	.174			

*Note. Durbin-Watson statistic = 2.02; Mean VIF = 1.05.*

## Discussion

Undergoing a traumatic amputation can significantly impact an individual’s HRQoL[2]. Gaining insight into the factors outside of that experience which can affect HRQoL can be beneficial in supporting the rehabilitation process. The aim of this study was to identify what factors may predict HRQoL in the upper limb amputee population. Based on the findings of this study, the ego-resilience trait, conceptualized here as a stable personality trait, as well as ethnicity are significant predicting factors for HRQoL after amputation and should be identified early in the care process.

By framing resilience as a stable trait (ego-resilience) rather than a transient process, these findings suggest that an individual’s baseline psychological flexibility may be a foundational determinant of their rehabilitative trajectory. This is particularly critical in upper limb prosthetic rehabilitation, which is characterized by a notoriously high rejection rate due to the steep learning curve and the physical and cognitive demands of device operation [28]. Individuals with higher baseline ego-resilience may possess the inherent flexibility required to persist through the frustrations of prosthetic training, whereas those with lower trait resilience may be at a higher risk for device abandonment and suboptimal HRQoL outcomes.

While trait resilience is generally considered less modifiable than process-based resilience, identifying deficits early allows for targeted clinical support. For instance, interventions focusing on cognitive-behavioral techniques or mindfulness can provide individuals with process strategies to supplement their baseline traits, potentially bridging the gap to a higher HRQoL [29, 30]. Ego-resilience and its relationship to HRQoL have been studied in a multitude of settings and populations, including individuals with significant diagnoses such as cancer, multiple sclerosis, HIV/AIDS, Parkinson’s Disease, and depression [7,11,14,31]. In many of these studies, a similar positive relationship has been identified, supporting the conclusion that higher ego-resilience may equate to higher perceived quality of life during or following a major health diagnosis or traumatic health-related event.

The relationship between ethnicity, ego-resilience and HRQoL has not been thoroughly studied. Relationships between ethnicity and QoL have been examined in some similar populations, however ethnicity is identified more as a contributor to other predicting factors, such as obesity or racial/ethnic discrimination [32–35]. Ethnicity may inform and be linked to the social and cultural coping mechanisms of the patient. While this relationship has not been studied in a similar population, a study on resilience during pregnancy by race and ethnicity determined that there is a difference in baseline resilience based on ethnicity and foreign-born status [36]. Based on the differing results of the multivariate linear regression analysis, further studies need to be conducted to identify how these factors relate to each other within the upper limb amputee population.

Furthermore, our findings regarding ethnicity as a predictor suggest that cultural and social contexts play a role in HRQoL, though the interaction between ethnicity and ego-resilience requires deeper investigation. The fact that traditional predictors like age and level of injury were not significant in this study contrasts with existing literature on lower limb populations, reinforcing the need for research dedicated specifically to the unique challenges of upper limb amputation [4,9].

### Limitations

Several limitations must be considered. This study utilized a convenience sample of 90 individuals, representing a subset of data from a larger clinical surveillance dataset gathered by Advanced Arm Dynamics between 2019 and 2025. While this provided access to a specialized population, the sample is heavily weighted toward digit (59%) and partial hand amputations (17.8%). While partial hand and finger level amputations are the most prevalent (approximately 90% of all upper limb amputations) this may limit the generalizability of these findings to individuals with more proximal amputations [37].

Additionally, the study was limited by a lack of ethnic diversity, with 81% of participants identifying as White. This imbalance may explain why ethnicity lost significance in the multivariate model despite appearing as a predictor in simple linear regression. Future research should utilize larger, stratified samples to more effectively examine how different amputation levels and diverse cultural backgrounds interact with trait ego-resilience to shape long-term recovery.

### Future research and implications

Future research should examine further the potential predictors for HRQoL for this population, utilizing more diverse and larger sample sizes. More in-depth tools could be used to create a better picture of the mental health status of these patients. Examining these factors longitudinally may also yield important relationships as they change over time. There could be demographic predictors, yet unidentified, which could be generalized to the wider population. Understanding the impacting factors can help to identify potential barriers to successful rehabilitation for this population.

## Conclusion

Based on the findings in this study, ego-resilience has been identified as a significant predicting factor for HRQoL. Higher ego-resilience score and trait likely will result in higher HRQoL scores. Therefore it is important to identify patients with lower ego-resilience to better support their rehabilitation and reintegration, both physically, psychologically and socially [12]. Understanding of how demographic variables, such as ethnicity, may directly or indirectly impact HRQoL can also be beneficial in the recovery process.

## Supporting information

S1 Data This dataset is representative of the data collected and used in analysis for this study. (XLSX)
