# Supplementary material for: Ego-resilience and health-related quality of life after acquired Upper Limb Amputation
Source: PLoS One. 2026 Jun 25;21(6):e0352208. doi: 10.1371/journal.pone.0352208 (PMC13298937; doi:10.1371/journal.pone.0352208)
Supplement: Attachment — Submitted filename: Response to Reviewers.docx [file pone.0352208.s003.docx]

**Response to Reviewers**

**Journal:** *PLOS One*

**Manuscript Title:** Ego-Resilience and Health-Related Quality of Life After Acquired Upper Limb Amputation

We would like to thank the reviewers for the thorough review of our revised manuscript and for catching the error in the Limitations section:

**Reviewer #2: Minor revision: Within limitations, you have listed that partial hand and digit amputations are 59%, however, it is 76.6%**

- We adjusted the sentence with the correct percentages (lines 323-4): “the sample is heavily weighted toward digit (59%) and partial hand amputations (17.8%)”
