# Supplementary material for: Ego-resilience and health-related quality of life after acquired Upper Limb Amputation
Source: PLoS One. 2026 Jun 25;21(6):e0352208. doi: 10.1371/journal.pone.0352208 (PMC13298937; doi:10.1371/journal.pone.0352208)
Supplement: Peer review history

# Peer review: Decision Letter 0

**DOI:** 10.1371/journal.pone.0352208.r001

**Authors:** Aliah Shaheen

3 Mar 2026

PONE-D-25-46989

Resilience and Quality of Life After Acquired Upper Limb Amputation

PLOS One

Dear Dr. Trent,

Thank you for submitting your manuscript to PLOS ONE. After careful consideration, we feel that it has merit but does not fully meet PLOS ONE’s publication criteria as it currently stands. Therefore, we invite you to submit a revised version of the manuscript that addresses the points raised during the review process.

Please submit your revised manuscript by Apr 17 2026 11:59PM. If you will need more time than this to complete your revisions, please reply to this message or contact the journal office at plosone@plos.org. When you're ready to submit your revision, log on to https://www.editorialmanager.com/pone/ and select the 'Submissions Needing Revision' folder to locate your manuscript file.

Please include the following items when submitting your revised manuscript:

- A letter that responds to each point raised by the academic editor and reviewer(s). You should upload this letter as a separate file labeled 'Response to Reviewers'.
- A marked-up copy of your manuscript that highlights changes made to the original version. You should upload this as a separate file labeled 'Revised Manuscript with Track Changes'.
- An unmarked version of your revised paper without tracked changes. You should upload this as a separate file labeled 'Manuscript'.

If you would like to make changes to your financial disclosure, please include your updated statement in your cover letter. Guidelines for resubmitting your figure files are available below the reviewer comments at the end of this letter.

If applicable, we recommend that you deposit your laboratory protocols in protocols.io to enhance the reproducibility of your results. Protocols.io assigns your protocol its own identifier (DOI) so that it can be cited independently in the future. For instructions see: https://journals.plos.org/plosone/s/submission-guidelines#loc-laboratory-protocols. Additionally, PLOS ONE offers an option for publishing peer-reviewed Lab Protocol articles, which describe protocols hosted on protocols.io. Read more information on sharing protocols at https://plos.org/protocols?utm_medium=editorial-email&utm_source=authorletters&utm_campaign=protocols.

We look forward to receiving your revised manuscript.

Kind regards,

Aliah Faisal Shaheen

Academic Editor

PLOS One

Journal Requirements:

When submitting your revision, we need you to address these additional requirements.

1.Please ensure that your manuscript meets PLOS ONE's style requirements, including those for file naming. The PLOS ONE style templates can be found at

https://journals.plos.org/plosone/s/file?id=wjVg/PLOSOne_formatting_sample_main_body.pdf and

https://journals.plos.org/plosone/s/file?id=ba62/PLOSOne_formatting_sample_title_authors_affiliations.pdf

2.  Please include captions for your Supporting Information files at the end of your manuscript, and update any in-text citations to match accordingly. Please see our Supporting Information guidelines for more information: http://journals.plos.org/plosone/s/supporting-information.

3. If the reviewer comments include a recommendation to cite specific previously published works, please review and evaluate these publications to determine whether they are relevant and should be cited. There is no requirement to cite these works unless the editor has indicated otherwise.

Additional Editor Comments:

The reviewers have raised concerns on some of the methodological choices and assumptions and the interpretation in the discussion. Please address the points raised below.

[Note: HTML markup is below. Please do not edit.]

Reviewer's Responses to Questions

**Comments to the Author**

1. Is the manuscript technically sound, and do the data support the conclusions?

The manuscript must describe a technically sound piece of scientific research with data that supports the conclusions. Experiments must have been conducted rigorously, with appropriate controls, replication, and sample sizes. The conclusions must be drawn appropriately based on the data presented.

Reviewer #1: Partly

Reviewer #2: Yes

**********

2. Has the statistical analysis been performed appropriately and rigorously?

Reviewer #1: I Don't Know

Reviewer #2: Yes

**********

3. Have the authors made all data underlying the findings in their manuscript fully available?

The PLOS Data policy requires authors to make all data underlying the findings described in their manuscript fully available without restriction, with rare exception (please refer to the Data Availability Statement in the manuscript PDF file). The data should be provided as part of the manuscript or its supporting information, or deposited to a public repository. For example, in addition to summary statistics, the data points behind means, medians and variance measures should be available. If there are restrictions on publicly sharing data—e.g. participant privacy or use of data from a third party—those must be specified.

Reviewer #1: Yes

Reviewer #2: Yes

**********

4. Is the manuscript presented in an intelligible fashion and written in standard English?

PLOS ONE does not copyedit accepted manuscripts, so the language in submitted articles must be clear, correct, and unambiguous. Any typographical or grammatical errors should be corrected at revision, so please note any specific errors here.

Reviewer #1: Yes

Reviewer #2: Yes

**********

5. Review Comments to the Author

Please use the space provided to explain your answers to the questions above. You may also include additional comments for the author, including concerns about dual publication, research ethics, or publication ethics. (Please upload your review as an attachment if it exceeds 20,000 characters)

Reviewer #1: The article addresses an interesting topic. It is suggested that the work be presented more effectively so that the results of the efforts are not wasted.

-The problem statement does not adequately express the necessity of the work.

-The methods section (statistical analysis) is ambiguous, and the reader does not clearly understand how the comparisons were made or how the groupings were structured.

-Why was the Spearman correlation coefficient used between two normal parameters?

-Please clarify that the assumptions of the regression are not violated.

-How was the sample size estimated?

-The diversity of the level of amputation is a concern, as nearly half of the samples were partial hand amputees. Did you consider a stratified sampline method?

-How did you get access to the Arm Dynamics outcome measures?

-The discussion provides a weak interpretation of the results, while it would be better also to mention issues such as the high rejection rate of prosthetics in the upper limb and other applications of the results in the ield.

Reviewer #2: Comments to the Author

This manuscript examines the relationship between Ego-Resilience and Health Related Quality of Life in individuals with upper limb loss. The topic is timely and clinically relevant, given the relative paucity of psychosocial research in upper limb loss comparative to lower limb loss populations The manuscript is well written and addresses an important construct that has meaningful impact for rehabilitation science and clinical practice.

My comments below are intended to strengthen conceptual clarity, improve precision in terminology, and enhance transparency for readers.

1. The manuscript would benefit from greater conceptual precision in its use of the term resilience. Currently, resilience and ego-resilience are used somewhat interchangeably. However, resilience is broadly conceptualized as the ability to adapt or respond to adversity, but there is no single consensus definition in the literature. Ego-Resilience, by contrast, refers more specifically to a personality trait reflecting dynamic flexibility in impulse control and adaptability. To avoid conflating constructs, I recommend the authors should use the word ego-resilience in the title and throughout the manuscript. Additionally, other literature that mentions previous knowledge about correlations with “resilience”, I suggest that the citations should be limited to trait resilience and not process resilience. This will help the reader to understand the difference between modifiable resilience (process resilience) and trait resilience (less modifiable). This refinement would strengthen the theoretical rigor of the paper and prevent conceptual ambiguity.

2. In the Plain English Summary, the authors could be more explicit when stating the implications of the findings. Currently it says, “Give a lot of helpful information to those ….” It would be helpful for the reader to know what that helpful information could be used for (i.e. suggestions for screening practices, interventions, directions for future research) Expanding the implications would enhance accessibility and impact non-academic audiences.

3. Page 3 line 63-64: The cited Amputee Coalition white paper is a valuable source. However, the statement that “17% of people are living with upper limb loss or limb difference” appears to misinterpret the report. The white paper indicates that 17% of annual limb amputations are upper limb amputations. I suggest that you consider removing the reference to upper limb difference, as this report does not describe persons with limb difference from birth.

4. Page 3 line 66-68: The rationale for focusing on upper limb populations is appropriate and important. However, the two cited studies do not directly compare upper and lower limb populations. If the intent is to demonstrate that most psychosocial or Health Related Quality of Life research has focused on lower limb loss, it may strengthen the argument to include a reference that examines upper and lower limb or state that existing literature is siloed by limb type. The point is more clearly made in the following paragraph, but minor refinement here would improve cohesion.

Minor suggestions:

1. The references for 3 and 5 are the same article, as well as 2 and 4. I am not sure this is aligned with Vancouver style citations. This could be revised for consistency with journal formatting requirements.

2. Including descriptive captions for figures and tables would improve readability and interpretability.

3. The manuscript references the Wellness Inventory and other measures that are not discussed further. If these variables are not central to the present analysis, it may be clearer to state that this study represents a subset of data from a larger clinical surveillance dataset, and not reference the other measures. This would reduce unnecessary detail and maintain focus.

**********

6. PLOS authors have the option to publish the peer review history of their article (what does this mean?). If published, this will include your full peer review and any attached files.

If you choose “no”, your identity will remain anonymous but your review may still be made public.

**Do you want your identity to be public for this peer review?**  For information about this choice, including consent withdrawal, please see our Privacy Policy.

Reviewer #1: No

Reviewer #2: No

**********

[NOTE: If reviewer comments were submitted as an attachment file, they will be attached to this email and accessible via the submission site. Please log into your account, locate the manuscript record, and check for the action link "View Attachments". If this link does not appear, there are no attachment files.]

To ensure your figures meet our technical requirements, please review our figure guidelines: https://journals.plos.org/plosone/s/figures

You may also use PLOS’s free figure tool, NAAS, to help you prepare publication quality figures: https://journals.plos.org/plosone/s/figures#loc-tools-for-figure-preparation.

NAAS will assess whether your figures meet our technical requirements by comparing each figure against our figure specifications.

# Author comment: Author response to Decision Letter 1

**DOI:** 10.1371/journal.pone.0352208.r002

14 May 2026

When submitting your revision, we need you to address these additional requirements.

1.Please ensure that your manuscript meets PLOS ONE's style requirements, including those for file naming.

These have been updated and corrected.

2. Please include captions for your Supporting Information files at the end of your manuscript, and update any in-text citations to match accordingly. Please see our Supporting Information guidelines for more information: http://journals.plos.org/plosone/s/supporting-information.

This has been added to the end of the manuscript.

3. If the reviewer comments include a recommendation to cite specific previously published works, please review and evaluate these publications to determine whether they are relevant and should be cited. There is no requirement to cite these works unless the editor has indicated otherwise.

Reviewer #1: The article addresses an interesting topic. It is suggested that the work be presented more effectively so that the results of the efforts are not wasted.

-The problem statement does not adequately express the necessity of the work.

• Thank you for this insight. We have revised the Introduction to more explicitly state the necessity of this research. Specifically, we have highlighted the siloed nature of existing Health-Related Quality of Life (HRQoL) research, which disproportionately focuses on lower limb populations, and linked the need for psychological predictors to the high rejection rates of upper limb prosthetics.

-The methods section (statistical analysis) is ambiguous, and the reader does not clearly understand how the comparisons were made or how the groupings were structured.

• We have clarified the structure of the statistical comparisons. The "Statistical Analysis" section now explicitly states that non-parametric tests (Kruskal-Wallis and Chi-square) were utilized for categorical demographic variables due to their non-normal distribution, while correlational and regression analyses were reserved for the continuous psychological parameters.

-Why was the Spearman correlation coefficient used between two normal parameters?

• Thank you for requesting this clarification. Spearman’s rho was initially selected to maintain methodological consistency across the study, as several demographic variables were non-normally distributed. However, to address the reviewer's concern regarding the normally distributed ego-resilience and HRQoL scores, we have added a post-hoc Pearson correlation result (r = 0.36, p < .001) to confirm the robustness of the relationship.

-Please clarify that the assumptions of the regression are not violated.

• Thank you for catching this oversight. We have added explicit confirmation of regression diagnostics. The manuscript now reports a Durbin-Watson statistic of 2.02, confirming the independence of residuals, and Variance Inflation Factor (VIF) values of 1.05, confirming the absence of multicollinearity.

-How was the sample size estimated?

• The manuscript now clarifies that a retrospective convenience sample of n = 90 was utilized, consisting of all available deidentified records meeting the inclusion criteria within the specific clinical surveillance window.

-The diversity of the level of amputation is a concern, as nearly half of the samples were partial hand amputees. Did you consider a stratified sampline method?

• We acknowledge the prevalence of digit and partial hand amputations in our sample (77%) as a limitation. We have updated the "Limitations" section to reflect that while these levels represent the majority of clinical upper limb cases epidemiologically (~90%), future research should utilize stratified sampling to better represent proximal amputation levels.

-How did you get access to the Arm Dynamics outcome measures?

• Thank you for catching this oversight. We have updated the "Participants" and "Competing Interests" sections to clarify that data were accessed through a research partnership and that the lead author facilitated the extraction of deidentified records from the organization's clinical surveillance dataset.

-The discussion provides a weak interpretation of the results, while it would be better also to mention issues such as the high rejection rate of prosthetics in the upper limb and other applications of the results in the ield.

• The Discussion has been expanded to link trait ego-resilience to the persistence required to overcome the steep learning curve and high rejection rates associated with upper limb prosthetics.

Reviewer #2: Comments to the Author

This manuscript examines the relationship between Ego-Resilience and Health Related Quality of Life in individuals with upper limb loss. The topic is timely and clinically relevant, given the relative paucity of psychosocial research in upper limb loss comparative to lower limb loss populations The manuscript is well written and addresses an important construct that has meaningful impact for rehabilitation science and clinical practice.

My comments below are intended to strengthen conceptual clarity, improve precision in terminology, and enhance transparency for readers.

1. The manuscript would benefit from greater conceptual precision in its use of the term resilience. Currently, resilience and ego-resilience are used somewhat interchangeably. However, resilience is broadly conceptualized as the ability to adapt or respond to adversity, but there is no single consensus definition in the literature. Ego-Resilience, by contrast, refers more specifically to a personality trait reflecting dynamic flexibility in impulse control and adaptability. To avoid conflating constructs, I recommend the authors should use the word ego-resilience in the title and throughout the manuscript. Additionally, other literature that mentions previous knowledge about correlations with “resilience”, I suggest that the citations should be limited to trait resilience and not process resilience. This will help the reader to understand the difference between modifiable resilience (process resilience) and trait resilience (less modifiable). This refinement would strengthen the theoretical rigor of the paper and prevent conceptual ambiguity.

• We appreciate this noted need for clarification and consistency. We have updated the title and the entire manuscript to use the term "ego-resilience" to reflect the trait-based nature of the ER89 scale. The Introduction now explicitly distinguishes between stable trait resilience and transient process-based resilience.

2. In the Plain English Summary, the authors could be more explicit when stating the implications of the findings. Currently it says, “Give a lot of helpful information to those ….” It would be helpful for the reader to know what that helpful information could be used for (i.e. suggestions for screening practices, interventions, directions for future research) Expanding the implications would enhance accessibility and impact non-academic audiences.

• The Plain English Summary has been rewritten as a cohesive paragraph that explicitly identifies implications for early clinical screening and the use of targeted interventions like mindfulness or cognitive-behavioral techniques.

3. Page 3 line 63-64: The cited Amputee Coalition white paper is a valuable source. However, the statement that “17% of people are living with upper limb loss or limb difference” appears to misinterpret the report. The white paper indicates that 17% of annual limb amputations are upper limb amputations. I suggest that you consider removing the reference to upper limb difference, as this report does not describe persons with limb difference from birth.

• Thank you for catching this misinterpretation. We have corrected the Introduction to state that 17% of annual amputations are upper limb and have removed the reference to "limb difference" to align strictly with the cited report on acquired amputation.

4. Page 3 line 66-68: The rationale for focusing on upper limb populations is appropriate and important. However, the two cited studies do not directly compare upper and lower limb populations. If the intent is to demonstrate that most psychosocial or Health Related Quality of Life research has focused on lower limb loss, it may strengthen the argument to include a reference that examines upper and lower limb or state that existing literature is siloed by limb type. The point is more clearly made in the following paragraph, but minor refinement here would improve cohesion.

• Thank you for this suggestion. We have refined the rationale to state that existing literature is often siloed by limb type, necessitating dedicated study into the unique psychosocial challenges of the upper limb population.

Minor suggestions:

1. The references for 3 and 5 are the same article, as well as 2 and 4. I am not sure this is aligned with Vancouver style citations. This could be revised for consistency with journal formatting requirements.

• Thank you for catching this! These have been updated appropriately to remove duplicate citations.

2. Including descriptive captions for figures and tables would improve readability and interpretability.

• Descriptive titles and captions have been added to all tables.

3. The manuscript references the Wellness Inventory and other measures that are not discussed further. If these variables are not central to the present analysis, it may be clearer to state that this study represents a subset of data from a larger clinical surveillance dataset, and not reference the other measures. This would reduce unnecessary detail and maintain focus.

• This is a great suggestion. The "Measures" section was streamlined to maintain focus on the primary variables of interest.

Attachment Submitted filename: Response to Reviewers.docx

# Peer review: Decision Letter 1

**DOI:** 10.1371/journal.pone.0352208.r003

**Authors:** Aliah Shaheen, Aliah Shaheen

3 Jun 2026

<div>PONE-D-25-46989R1

Ego-Resilience and Health Related Quality of Life after acquired upper limb amputation

PLOS One

Dear Dr. Trent,

Thank you for submitting your manuscript to PLOS ONE. After careful consideration, we feel that it has merit but does not fully meet PLOS ONE’s publication criteria as it currently stands. Therefore, we invite you to submit a revised version of the manuscript that addresses the points raised during the review process.

Please review the very minor typo outlined by the reviewer.

Please submit your revised manuscript by Jul 18 2026 11:59PM. If you will need more time than this to complete your revisions, please reply to this message or contact the journal office at plosone@plos.org. When you're ready to submit your revision, log on to https://www.editorialmanager.com/pone/ and select the 'Submissions Needing Revision' folder to locate your manuscript file.

Please include the following items when submitting your revised manuscript:

- A letter that responds to each point raised by the academic editor and reviewer(s). You should upload this letter as a separate file labeled 'Response to Reviewers'.
- A marked-up copy of your manuscript that highlights changes made to the original version. You should upload this as a separate file labeled 'Revised Manuscript with Track Changes'.
- An unmarked version of your revised paper without tracked changes. You should upload this as a separate file labeled 'Manuscript'.

If you would like to make changes to your financial disclosure, please include your updated statement in your cover letter. Guidelines for resubmitting your figure files are available below the reviewer comments at the end of this letter.

If applicable, we recommend that you deposit your laboratory protocols in protocols.io to enhance the reproducibility of your results. Protocols.io assigns your protocol its own identifier (DOI) so that it can be cited independently in the future. For instructions see: https://journals.plos.org/plosone/s/submission-guidelines#loc-laboratory-protocols. Additionally, PLOS ONE offers an option for publishing peer-reviewed Lab Protocol articles, which describe protocols hosted on protocols.io. Read more information on sharing protocols at https://plos.org/protocols?utm_medium=editorial-email&utm_source=authorletters&utm_campaign=protocols.

As the corresponding author, your ORCID iD is verified in the submission system and will appear in the published article. PLOS supports the use of ORCID, and we encourage all coauthors to register for an ORCID iD and use it as well. Please encourage your coauthors to verify their ORCID iD within the submission system before final acceptance, as unverified ORCID iDs will not appear in the published article. *Only*  the individual author can complete the verification step; PLOS staff *cannot*  verify ORCID iDs on behalf of authors.

We look forward to receiving your revised manuscript.

Kind regards,

Aliah Faisal Shaheen

Academic Editor

PLOS One

Journal Requirements:

If the reviewer comments include a recommendation to cite specific previously published works, please review and evaluate these publications to determine whether they are relevant and should be cited. There is no requirement to cite these works unless the editor has indicated otherwise.

Please review your reference list to ensure that it is complete and correct. If you have cited papers that have been retracted, please include the rationale for doing so in the manuscript text, or remove these references and replace them with relevant current references. Any changes to the reference list should be mentioned in the rebuttal letter that accompanies your revised manuscript. If you need to cite a retracted article, indicate the article’s retracted status in the References list and also include a citation and full reference for the retraction notice.

[Note: HTML markup is below. Please do not edit.]

Reviewers' comments:

Reviewer's Responses to Questions

**Comments to the Author**

1. If the authors have adequately addressed your comments raised in a previous round of review and you feel that this manuscript is now acceptable for publication, you may indicate that here to bypass the “Comments to the Author” section, enter your conflict of interest statement in the “Confidential to Editor” section, and submit your "Accept" recommendation.

Reviewer #2: (No Response)

**********

2. Is the manuscript technically sound, and do the data support the conclusions?

The manuscript must describe a technically sound piece of scientific research with data that supports the conclusions. Experiments must have been conducted rigorously, with appropriate controls, replication, and sample sizes. The conclusions must be drawn appropriately based on the data presented.

Reviewer #2: Yes

**********

3. Has the statistical analysis been performed appropriately and rigorously?

Reviewer #2: Yes

**********

4. Have the authors made all data underlying the findings in their manuscript fully available?

The PLOS Data policy requires authors to make all data underlying the findings described in their manuscript fully available without restriction, with rare exception (please refer to the Data Availability Statement in the manuscript PDF file). The data should be provided as part of the manuscript or its supporting information, or deposited to a public repository. For example, in addition to summary statistics, the data points behind means, medians and variance measures should be available. If there are restrictions on publicly sharing data—e.g. participant privacy or use of data from a third party—those must be specified.

Reviewer #2: Yes

**********

5. Is the manuscript presented in an intelligible fashion and written in standard English?

PLOS ONE does not copyedit accepted manuscripts, so the language in submitted articles must be clear, correct, and unambiguous. Any typographical or grammatical errors should be corrected at revision, so please note any specific errors here.

Reviewer #2: Yes

**********

6. Review Comments to the Author

Please use the space provided to explain your answers to the questions above. You may also include additional comments for the author, including concerns about dual publication, research ethics, or publication ethics. (Please upload your review as an attachment if it exceeds 20,000 characters)

Reviewer #2: Minor revision: Within limitations, you have listed that partial hand and digit amputations are 59%, however, it is 76.6%

**********

7. PLOS authors have the option to publish the peer review history of their article (what does this mean?). If published, this will include your full peer review and any attached files.

If you choose “no”, your identity will remain anonymous but your review may still be made public.

**Do you want your identity to be public for this peer review?**  For information about this choice, including consent withdrawal, please see our Privacy Policy.

Reviewer #2: No

**********

[NOTE: If reviewer comments were submitted as an attachment file, they will be attached to this email and accessible via the submission site. Please log into your account, locate the manuscript record, and check for the action link "View Attachments". If this link does not appear, there are no attachment files.]

To ensure your figures meet our technical requirements, please review our figure guidelines: https://journals.plos.org/plosone/s/figures

You may also use PLOS’s free figure tool, NAAS, to help you prepare publication quality figures: https://journals.plos.org/plosone/s/figures#loc-tools-for-figure-preparation.

NAAS will assess whether your figures meet our technical requirements by comparing each figure against our figure specifications.

# Author comment: Author response to Decision Letter 2

**DOI:** 10.1371/journal.pone.0352208.r004

3 Jun 2026

We would like to thank the reviewers for the thorough review of our revised manuscript and for catching the error in the Limitations section:

Reviewer #2: Minor revision: Within limitations, you have listed that partial hand and digit amputations are 59%, however, it is 76.6%

• We adjusted the sentence with the correct percentages (lines 323-4): “the sample is heavily weighted toward digit (59%) and partial hand amputations (17.8%)”

Attachment Submitted filename: Response to Reviewers v3.docx

# Editor report: Decision Letter 2

**DOI:** 10.1371/journal.pone.0352208.r005

**Authors:** Aliah Shaheen, Aliah Shaheen, Aliah Shaheen

7 Jun 2026

Ego-Resilience and Health Related Quality of Life after acquired upper limb amputation

PONE-D-25-46989R2

Dear Dr. Trent,

We’re pleased to inform you that your manuscript has been judged scientifically suitable for publication and will be formally accepted for publication once it meets all outstanding technical requirements.

Within one week, you’ll receive an e-mail detailing the required amendments. When these have been addressed, you’ll receive a formal acceptance letter and your manuscript will be scheduled for publication.

An invoice will be generated when your article is formally accepted. Please note, if your institution has a publishing partnership with PLOS and your article meets the relevant criteria, all or part of your publication costs will be covered. Please make sure your user information is up-to-date by logging into Editorial Manager at Editorial Manager® and clicking the ‘Update My Information' link at the top of the page. For questions related to billing, please contact billing support.

If your institution or institutions have a press office, please notify them about your upcoming paper to help maximize its impact. If they’ll be preparing press materials, please inform our press team as soon as possible -- no later than 48 hours after receiving the formal acceptance. Your manuscript will remain under strict press embargo until 2 pm Eastern Time on the date of publication. For more information, please contact onepress@plos.org.

Kind regards,

Aliah Faisal Shaheen

Academic Editor

PLOS One

Additional Editor Comments (optional):

Reviewers' comments:

# Editor report: Acceptance letter

**DOI:** 10.1371/journal.pone.0352208.r006

**Authors:** Aliah Shaheen, Aliah Shaheen, Aliah Shaheen

PONE-D-25-46989R2

PLOS One

Dear Dr. Trent,

I'm pleased to inform you that your manuscript has been deemed suitable for publication in PLOS One. Congratulations! Your manuscript is now being handed over to our production team.

At this stage, our production department will prepare your paper for publication. This includes ensuring the following:

* All references, tables, and figures are properly cited

* All relevant supporting information is included in the manuscript submission,

* There are no issues that prevent the paper from being properly typeset

You will receive further instructions from the production team, including instructions on how to review your proof when it is ready. Please keep in mind that we are working through a large volume of accepted articles, so please give us a few days to review your paper and let you know the next and final steps.

Lastly, if your institution or institutions have a press office, please let them know about your upcoming paper now to help maximize its impact. If they'll be preparing press materials, please inform our press team within the next 48 hours. Your manuscript will remain under strict press embargo until 2 pm Eastern Time on the date of publication. For more information, please contact onepress@plos.org.

You will receive an invoice from PLOS for your publication fee after your manuscript has reached the completed accept phase. If you receive an email requesting payment before acceptance or for any other service, this may be a phishing scheme. Learn how to identify phishing emails and protect your accounts at https://explore.plos.org/phishing.

If we can help with anything else, please email us at customercare@plos.org.

Thank you for submitting your work to PLOS ONE and supporting open access.

Kind regards,

PLOS ONE Editorial Office Staff

on behalf of

Dr. Aliah Faisal Shaheen

Academic Editor

PLOS One
